# Cytotoxic Effects of Biosynthesized Zinc Oxide Nanoparticles on Murine Cell Lines

**DOI:** 10.1155/2015/593014

**Published:** 2015-02-16

**Authors:** Farideh Namvar, Heshu Sulaiman Rahman, Rosfarizan Mohamad, Susan Azizi, Paridah Mohd Tahir, Max Stanley Chartrand, Swee Keong Yeap

**Affiliations:** ^1^Institute of Tropical Forestry and Forest Products (INTROP), Universiti Putra Malaysia (UPM), 43400 Serdang, Selangor, Malaysia; ^2^Research Center for Animal Development Applied Biology, Mashhad Branch, Islamic Azad University, Mashhad, Iran; ^3^Department of Clinic and Internal Medicine, College of Veterinary Medicine, University of Sulaimani, Sulaimani Nwe, Street 27, Sulaimani City, Kurdistan Region, Iraq; ^4^Department of Veterinary Laboratory Diagnosis, Faculty of Veterinary Medicine, Universiti Putra Malaysia (UPM), 43400 Serdang, Selangor, Malaysia; ^5^Institute of Bioscience (IBS), Universiti Putra Malaysia (UPM), 43400 Serdang, Selangor, Malaysia; ^6^Department of Bioprocess Technology, Faculty of Biotechnology and Biomolecular Sciences, Universiti Putra Malaysia (UPM), 43400 Serdang, Selangor, Malaysia; ^7^DigiCare Behavioral Research, Casa Grande, AZ, USA

## Abstract

The aim of this study is to evaluate the *in vitro* cytotoxic activity and cellular effects of previously prepared ZnO-NPs on murine cancer cell lines using brown seaweed (*Sargassum muticum*) aqueous extract. Treated cancer cells with ZnO-NPs for 72 hours demonstrated various levels of cytotoxicity based on calculated IC_50_ values using MTT assay as follows: 21.7 ± 1.3 *μ*g/mL (4T1), 17.45 ± 1.1 *μ*g/mL (CRL-1451), 11.75 ± 0.8 *μ*g/mL (CT-26), and 5.6 ± 0.55 *μ*g/mL (WEHI-3B), respectively. On the other hand, ZnO-NPs treatments for 72 hours showed no toxicity against normal mouse fibroblast (3T3) cell line. On the other hand, paclitaxel, which imposed an inhibitory effect on WEHI-3B cells with IC_50_ of 2.25 ± 0.4, 1.17 ± 0.5, and 1.6 ± 0.09 *μ*g/mL after 24, 48, and 72 hours treatment, respectively, was used as positive control. Furthermore, distinct morphological changes were found by utilizing fluorescent dyes; apoptotic population was increased via flowcytometry, while a cell cycle block and stimulation of apoptotic proteins were also observed. Additionally, the present study showed that the caspase activations contributed to ZnO-NPs triggered apoptotic death in WEHI-3 cells. Thus, the nature of biosynthesis and the therapeutic potential of ZnO-NPs could prepare the way for further research on the design of green synthesis therapeutic agents, particularly in nanomedicine, for the treatment of cancer.

## 1. Introduction

Nanoscience and nanotechnology have excellent potential in a broad spectrum of cancer research, such as in diagnostics, monitoring, and therapeutic strategies [1, 2]. Some nanocarriers like liposome, dendrimer, micelle, carbon nanotube, and nanoparticles have attempted in the diagnosis and theranostics of several types of cancers [3, 4]. The Green approach for synthesis nanoparticles, using plant materials [5] for reducing and capping agents, could be quite attractive for use in nanobiotechnology [6, 7]. This technology, as compared to other mechanical strategies, is safe, simple, nontoxic, efficient, environmentally friendly, and efficacious as single-potreactions, without the need for additional surfactants and capping agents [2, 8].

Zinc oxide, due to physical and chemical properties, is considered a capable agent in cancer therapy [9]. A novel approach of biosynthesis, zinc oxide nanoparticles (ZnO-NPs) produced nanoparticles using a natural source, such as plant extracts to reduce metal ions, which are readily scalable and nontoxic compared with physical and chemical methods [10].

The mechanisms of cytotoxicity from ZnO-NPs are not yet entirely understood, but the generation of hydroxyl radicals (OH^•^), superoxide anion (O_2_^−^), and perhydroxyl radicals (HO_2_^•^) from the surface of ZnO are believed to be major components. When nanoparticles interact with cells, cellular protection mechanisms are activated to minimize harm. However, if the highly active free radicals production exceeds the antioxidative defensive ability of the cell, it results in oxidative harm of biomolecules which can lead to cell death [11, 12]. Recent studies have confirmed that bioactive compounds obtained from macroalgae provide an opportunity for discovering new pharmaceutical and versatile novel agents with more promise in cancer research, diagnostic and efficient treatment [13].

As mentioned in previous reports [14], seaweed as a subgroup of macroalgae is an available food source that is consumed in many countries, most traditionally in southeast Asia [15]. Seaweed potentially has biologically active substrates like polysaccharides, proteins, lipid, vitamin, soluble fiber, and minerals with multiple medicinal applications against cancer [16], inflammation [17], allergy [18], diabetes [19], thrombosis [20], reduction of obesity by bringing down the caloric value of the diet [21], reduction of lipid absorption and cardiovascular diseases [22], hypertension [23], and other degenerative chronic diseases [24]. These biomedical applications are mainly due to existing functional groups, which act as a capping agent in a Green single step process. Polysaccharides are the main constituents of biopolymers in seaweed water extract and were found to be strong stabilizers, for increased biocompatibility, conferring chemical functionality towards nanostructures including iron oxide magnetic nanoparticles [25].

In the previous study [26], we synthesized and characterized zinc oxide nanoparticles (ZnO-NPs) using* Sargassum muticum*, brown seaweed extract in green method. The aim of this study is in evaluating the cytotoxic effect of ZnO-NPs prepared via biosynthesize green method on various human cancer cells using different experimental methods.

## 2. Material and Methods

### 2.1. Materials

Samples of the* Sargassum muticum*, brown seaweed, were collected from the waters of Persian Gulf coastal areas. All aqueous solutions were prepared using distilled water.

### 2.2. Cell Lines and Cell Culture Conditions

All murine cancer cell lines (CT-26, WEHI-3, 4T1, and CRL-1451) and normal mice fibroblast cell line (3T3) were purchased from the American Type Culture Collection (ATCC) (Maryland, USA). All cell lines (except CRL-1451) were cultured in RPMI-1640 (GIBCO, Germany) medium, supplemented with 10% (v/v) fetal bovine serum (FBS) (GIBCO, Germany) and 1% penicillin/streptomycin (GIBCO, Germany) (100 units/mL penicillin and 50 *μ*g/mL streptomycin) according to the supplier protocol, whereas ATCC-formulated Dulbecco's Modified Eagle's Medium (DMEM) was used as a base medium for CRL-1451 cell line with same supplementation mentioned before. Cell cultures were incubated in a humidified atmosphere of 95% air and 5% carbon dioxide at 37°C.

### 2.3. Preparation of (*Sargassum muticum*) Extract

As in a previous study [26], for the preparation of extract, about 2.0 g of seaweed specimens was ground, freeze-dried, and boiled in 100 mL of double distilled water with continuous stirring for 15 minutes. Then, the extract was left at 25°C, filtered, and then stored at −20°C for further investigation.

### 2.4. Preparation of ZnO-NPs

Zinc acetate dehydrate (Zn(Ac)_2_2H_2_O) (2 mM) solution was made to react with 50 mL of the aqueous extract for 3-4 hours in an aqueous bath (Falc MF24, Progen scientific) system under continuous stirring at 70°C. The pale white solid product was collected through centrifugation at 4000 rpm (Avanti J25, Beckman) for 10 minutes and carefully washed with distilled water and then dried at 100°C overnight [26].

### 2.5. Cell Viability Assay

Cell viability of various cancer cell lines and normal cell line treated with ZnO-NPs were assessed with 3-(4,5-dimethylthiazol-2-yl)-2,5-diphenyltetrazolium bromide (MTT, Sigma Aldrich, USA). Murine cancer and noncancer cells were seeded at a density of 2 × 10^5^ cells/well in 96-well microplates for 24 hours. Various concentrations (1–100 *μ*g) of ZnO-NPs in medium were prepared and added to the cultured cells. After 72 hours of incubation, 20 *μ*L of the freshly prepared MTT solution was added to each well. After incubation for 4 hours at 37°C, cell viability was measured spectrophotometrically at 570 nm (Microplate reader, Biotech Inc., USA). The concentration, which inhibited 50% of cellular growth (IC_50_ value), was determined and calculated by the following formula:

(1) $$\begin{array}{c} \text{Growth}\;\;\text{inhibition}={\text{OD}}_{\text{control}}-{\text{OD}}_{\text{treated}\;\;\text{sample}}\times \frac{100}{\text{O}{\text{D}}_{\text{control}}}. \end{array}$$

The cytotoxicity of ZnO-NPs on cells was expressed as IC_50_ values (the drug concentration, reducing the absorbance of treated cells by 50% with respect to untreated cells). This experiment was carried out in triplicate. The DMSO (0.1%) was used as negative control.

### 2.6. Cellular Morphology Assay Using AO/PI Double Staining

Acridine orange/propidium iodide (AO/PI) double staining was used to check the ZnO-NPs-induced morphological alterations in murine myelocytic leukemia cells. Briefly, the WEHI-3B cells were plated at a concentration of 1 × 10^6^ cells/mL in a 25 cm^2^ culture flask (TPP, Switzerland) and then treated with IC_50_ of ZnO-NPs and incubated at 37°C in a 5% carbon dioxide incubator for 24, 48, and 72 hours, respectively. Then, cells were trypsinized, centrifuged, and washed with ice-cold PBS and stained with AO/PI (1 : 1 v/v) at 100 *μ*g/mL. Cover slip fixed slides with antifade between them were observed under a fluorescence microscope (Leica, Japan) in the dark with the Q-floro software installed.

### 2.7. Measuring of Apoptotic Cells Using Annexin V-TITC

The effect of apoptosis was flow cytometrically measured using an annexin V-FITC apoptosis detection kit (Sigma Aldrich, USA). In brief, the WEHI-3 cells (1 × 10^6^ cells/mL) were exposed to nanoparticles for 12, 24, and 48 hours, while untreated cells were used as controls. After that, both floating and attached cells were harvested, washed in prechilled PBS, centrifuged, and resuspended in 1x binding buffer. Then, the cells were double stained with ice in the dark for 15 min with the fluorescein isothiocyanate- (FITC-) labeled annexin V (5 *μ*L) and PI (10 *μ*L) before being analyzed flow cytometrically using FACSCalibur (BD, USA). Data analysis was performed using the Cell Quest Pro software.

### 2.8. Analysis of DNA Content by Flow Cytometry

Flow cytometer was used to support the cytotoxicity of ZnO-NPs towards WEHI-3 cells. Briefly, The WEHI-3 cells 2.0 × 10^6^ cells/mL were cultured with the IC_50_ of ZnO-NPs and incubated for 24, 48, and 72 hours, respectively. WEHI-3 cells were trypsinized and washed twice with ice-cold PBS. Cells were stored in 600 *μ*L 70% ethanol at −20°C. The next day, the cells were centrifuged, washed twice with prechilled PBS, and incubated with 1 mL staining buffer containing 1% Triton X-100, 10 *μ*g RNase A, and 50 *μ*g/mL propidium iodide for 20 min in the dark. Then, the samples were analyzed using FACSCalibur flow cytometry (BD, USA). Data analysis was performed using the Cell Quest Pro software.

### 2.9. Caspase Protease Activity Assay Using a Microplate Reader

To search for the mechanisms involved in apoptosis, the protease activities of caspase-3 and caspase-9 in exponentially growing WEHI-3 cells treated with ZnO-NPs were determined respectively, using a colorimetric assay kit (Gene script kit, Piscataway, NJ 08854, USA) according to the instructions of the manufacturing company without any modification. Briefly, WEHI-3 cells (1 × 10^6^ cells) were pretreated with ZnO-NPs for 24, 48, and 72 hours, respectively. Then, the cells were collected and washed with prechilled PBS and cell lysates were prepared in a 100 *μ*L lysis buffer for 20 minutes on ice. After centrifugation, the supernatants were collected and the total protein was quantified by the Bradford assay. Lastly, the lysates were incubated at 37°C for 4 hours in the dark and the absorption at 490 nm was measured using an ELISA microplate reader (Biotech Inc., USA).

### 2.10. Whole Cell Extract Preparation and Immunoblot

Immunoblotting was conducted according to the earlier study [27]. Briefly, WEHI-3 cells were grown in 75 cm^2^ culture flask and then treated with ZnO-NPs. After incubation for 24, 48, and 72 hours, respectively, harvested cells were washed twice with ice-cold PBS. Then, total cell proteins were isolated using a RIPA lysis buffer, separated on an SDS-PAGE, and transferred to PVDF membranes. After being blocked, the membranes were incubated with anti-Bcl-2 (Santa Cruz, CA, USA) and anti-Bax (Santa Cruz, CA, USA) (1 : 1000) at 4°C overnight. After washing for 30 minutes at 10 minute intervals, the membranes were loaded with an HRP secondary antibody (Goat polyclonal to rabbit IgG, AB97051, Abcam, USA) (1 : 2000) at room temperature for 1 hour. Finally, specific protein bands were detected using chemiluminescence (ECL) detection kit (Abcam, USA). *β*-actin was used as the internal control (Santa Cruz, CA, USA).

### 2.11. Statistical Analysis

The assays were performed in triplicate, and the results were expressed as mean ± SD. The statistical analysis was done using SPSS version 20.0 (SPSS Inc., Chicago, USA). Probability values of less than 0.05 (*P* < 0.05) were considered statistically significant.

## 3. Results and Discussion

### 3.1. Biosynthesis of ZnO-NPs Using Brown Seaweed Extract

**Figure 1:**
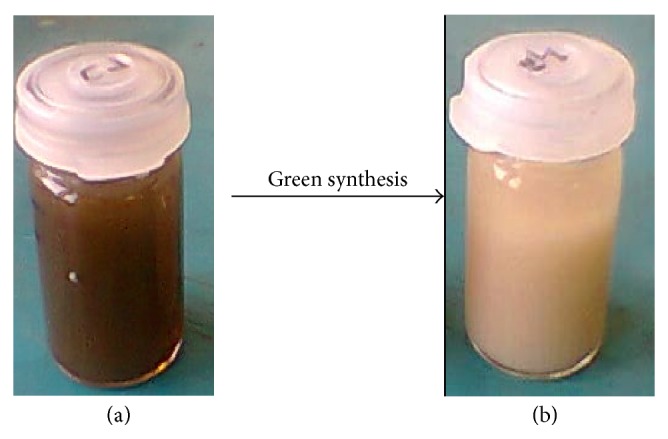
The aqueous extract of* S. muticum* (a) before and (b) after synthesis of ZnO-NPs.

In the previous investigation [26], ZnO-NPs were prepared using* Sargassum muticum* aqueous extract by the green synthesis approach, which is more reliable and less toxic when compared to other methods. For Green biosynthesis of ZnO-NPs, Zinc acetate dehydrate solution was added to the brown seaweed extract containing sulfated polysaccharides with functional groups that led to the formation and stabilization of the ZnO nanoparticles. The formation of ZnO nanoparticles during the reaction was confirmed visually, as the brownish color of the mixture turned to a pale white color within 3 hours of preparation, indicating the synthesis of ZnO nanoparticles was successful (Figure 1).

Nagarajan and Arumugam Kuppusamy [28] reported biosynthesis of zinc oxide nanoparticles using various seaweeds such as green (*Caulerpa peltata*), red (*Hypnea Valencia*), and brown (*Sargassum myriocystum*). The preliminary screening of physicochemical parameters revealed that one seaweed* S. myriocystum* was able to synthesize zinc oxide nanoparticles. It was confirmed through the initial color change of the reaction mixture and via UV visible spectrophotometer. Based on their FTIR results, fucoidan water soluble pigments present in* S. myriocystum* leaf extract were responsible for reduction and stabilization of zinc oxide nanoparticles [28]. Jegan and Ramasubbu [29] also reported a novel agar-zinc oxide nanostructure. The morphological observation of the SEM results revealed that the ZnO nanostructures were between 50 and 100 nm in size and embedded in the agar matrix.

### 3.2. Characterizations of the Synthesized Zinc Oxide NPs

**Figure 2:**
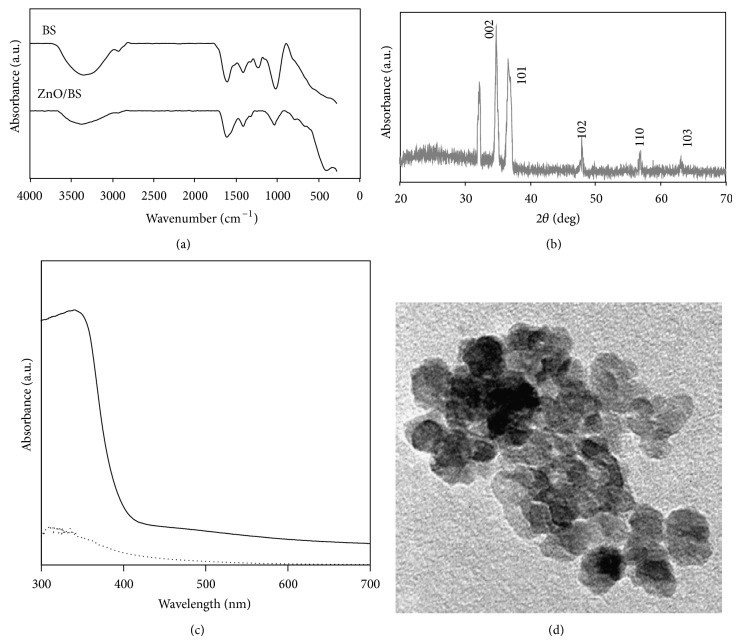
(a) FTIR spectra and (b) XRD and (c) UV-visible and (d) TEM image of biosynthesized ZnO-NPs.

Prepared ZnO-NPs were characterized with FTIR spectroscopy, X-ray diffraction, UV-visible, and transmission electron microscope (TEM) observations. FTIR spectra showed the sulfate and hydroxyl moieties of polysaccharide in the formation of ZnO-NPs. X-ray diffraction was recognized with the crystalline structure and phase purity of the ZnO-NPs. UV-visible A showed a sharp absorption in the wavelength of 334 nm clarified the basic band gap absorption of ZnO crystals. TEM observation indicated that ZnO-NPs had hexagonal wurtzite structure and that the average size ranged from 10 nm to 15 nm (Figure 2).

### 3.3. ZnO-NPs Inhibits Proliferation of Murine Cancer Cells

**Figure 3:**
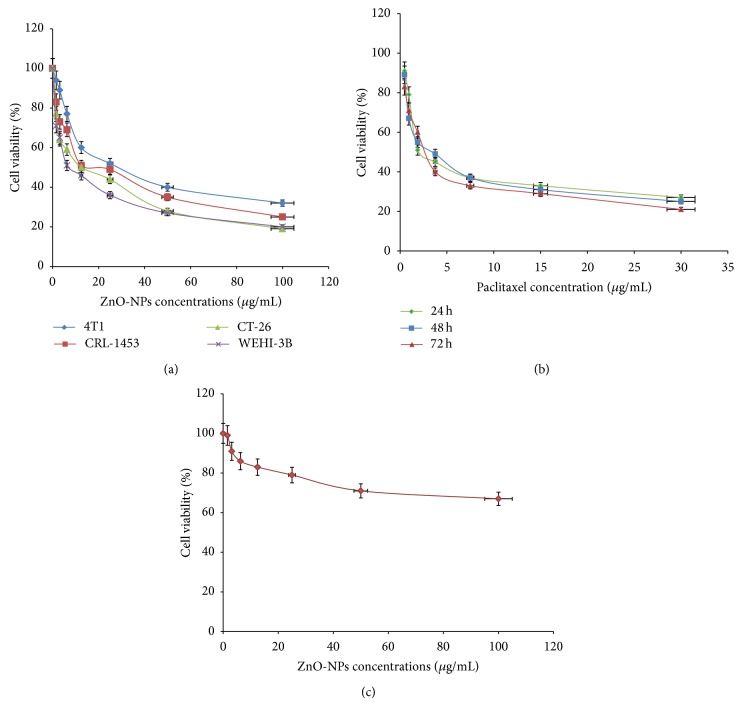
(a) Cytotoxic effect of ZnO-NPs on various cancer cells at 72 h of treatment was evaluated through mitochondrial activity using the MTT assay. Each point is the mean value of three replicates. (b) Cytotoxic effects of paclitaxel on WEHI-3B cells at 24, 48, and 72 h of treatment were evaluated through mitochondrial activity using the MTT assay. Each point is the mean value of three replicates. (c) Cytotoxic effects of ZnO-NPs on normal mouse fibroblast cell line (3T3) at 72 h of treatment were evaluated with MTT assay. Each point is the mean value of three replicates.

To examine the cytotoxicity effect of ZnO-NPs* in vitro*, various murine cancer cells and normal murine fibroblast cells were incubated with various concentrations of ZnO-NPs. The antiproliferative effect was determined using MTT assay, which is considered more reliable [30]. The results showed that ZnO-NPs dose and time-dependently significantly (*P* < 0.05) inhibited the proliferation of the various cancer cell lines (Figure 3(a)). The IC_50_ values calculated for ZnO-NPs on cells were 21.7 ± 1.3 *μ*g/mL (4T1), 17.45 ± 1.1 *μ*g/mL (CRL-1451), 11.75 ± 0.8 *μ*g/mL (CT-26), and 5.6 ± 0.55 *μ*g/mL (WEHI-3) after treatment for 72 hours. Thus, the WEHI-3B cell line was used for further investigations. Paclitaxel a control positive drug and drug of choice for treating leukemia imposed an inhibitory effect on WEHI-3B cells with IC_50_ of 2.25 ± 0.4, 1.17 ± 0.5, and 1.6 ± 0.09 *μ*g/mL after 24, 48, and 72 hours treatment, respectively (Figure 3(b)). On the other hand, ZnO-NPs did not show any toxic effect on normal fibroblast cell line (Figure 3(c)).

Relevant to the evaluation of toxicity of metal nanoparticles against many animals, cancer cells have been reported. Akhtar et al. revealed that three types of cancer cells were killed by the effect of ZnO-NPs, while normal rat astrocytes and hepatocytes were not affected [31].

### 3.4. Morphological Changes Induced by ZnO-NPs in Leukaemia Cells

**Figure 4:**
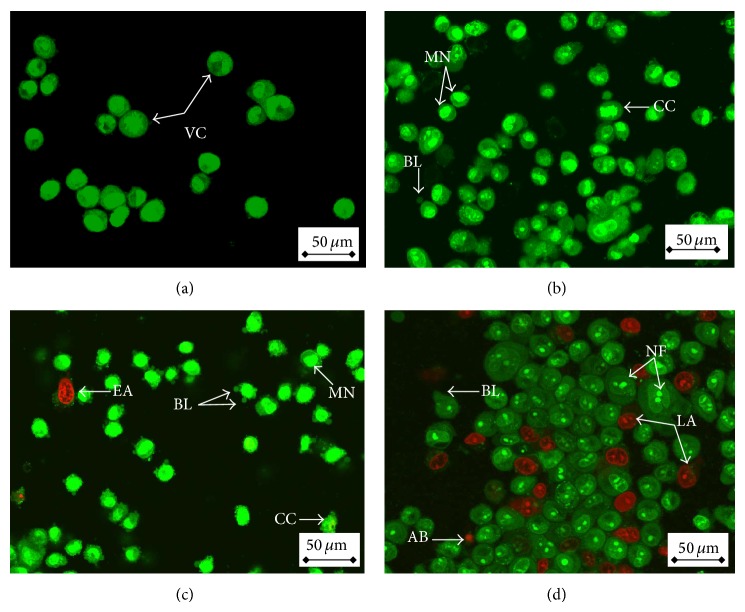
Fluorescent micrograph of AO/PI double stained WEHI-3B cells that was treated with ZnO-NPs. (a) Untreated cells showing normal cell structure. (b) Early apoptotic cells after 24 h treatment showing membrane blebbing and chromatin condensation. (c) Blebbing and nuclear margination after 48 h treatment. (d) DNA fragmentation and apoptotic body formation after 72 h treatment. VC: viable cells; EA: early apoptotic cells; CC: chromatin condensation; BL: blebbing; MN: marginated nucleus; FN: fragmented nucleus; LA: late apoptotic cells; and AB: apoptotic body.

To confirm whether the growth inhibition of ZnO-NPs was caused by apoptosis, annexin V-FITC/PI double staining was analyzed. After WEHI-3 cells were incubated with ZnO-NPs for different time periods, it was demonstrated that the death of WEHI-3 cells indeed occurred via apoptosis. Thus, the treated cells were characterized by membrane blebbing, nuclear condensation, nuclear fragmentation, and apoptotic body formation. These abnormal cell features could be regarded as a morphological symbol of apoptosis [32]. Simultaneously, there were no morphological changes found in the control group. The results obtained in our study indicated that ZnO-NPs initiated and provoked apoptosis in WEHI-3 cells (Figure 4).

Excluding of the PI and uptaking of AO by intact cell membranes allowed double stranded DNA to show green fluoresces under 488 nm excitation. On the other hand, apoptotic cells with condensed chromatin and affected cell membrane, which resulted in clumps of intense green fluorescent spots within the cell. Similar results to this current study were found by Rahman et al. and Namvar et al. [33, 34].

### 3.5. Effect on Annexin V-FITC Binding

**Figure 5:**
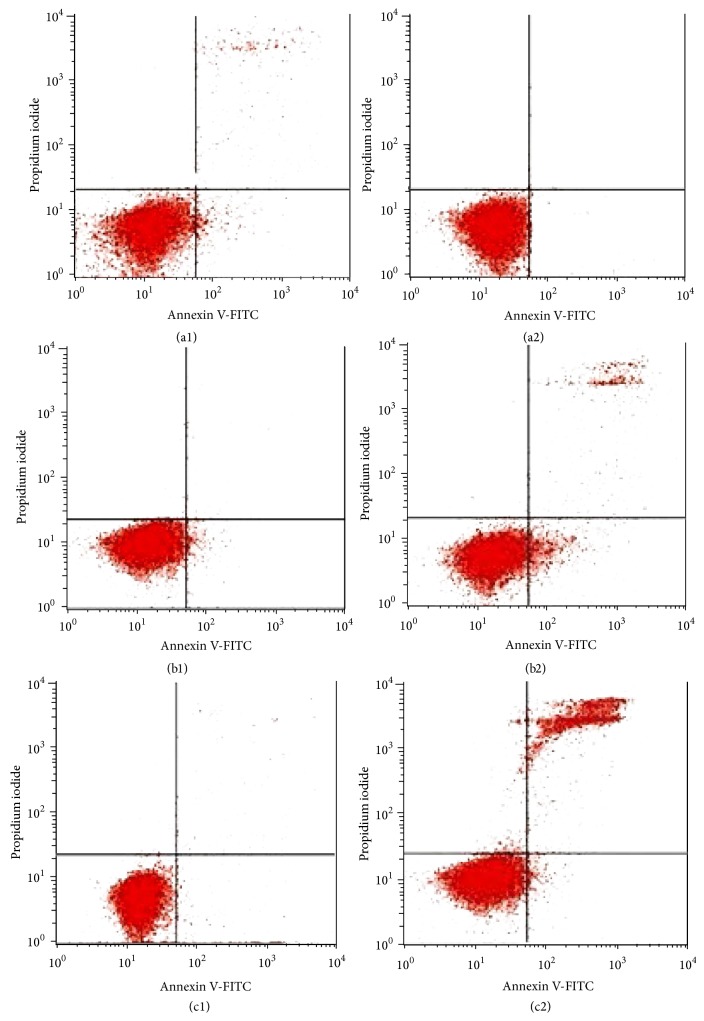
Flow cytometric analysis of apoptosis induction by ZnO-NPs in WEHI-3B cells after staining with FITC-conjugated annexin V and PI. (a1)–(c1) untreated (control) WEHI-3B cells at 12, 24, and 48 h incubation, respectively. (a2)–(c2) effects of 12, 24, and 48 h ZnO-NPs treatment, respectively.

The apoptotic effect of induction of ZnO-NPs was further confirmed by the evaluation of the number of apoptotic cells using flow cytometric analysis with the AV/PI double staining. Generally, an early event in apoptosis is started with translocation of phosphatidylserine to the outer part of plasma membrane from the inner part. In the presence of calcium ions, binding of annexin V to phosphatidylserines results in green fluorescence. These results increased membrane permeability during late apoptosis or necrosis leading to entrance of PI into cells as well that binds to cellular DNA and stains the nucleus red. Results in our study showed that ZnO-NPs treatment yielded a significant (*P* < 0.05) rise in the percentage of annexin V-FITC and PI positive cells (upper right quadrant) in a time dependent manner, indicating late apoptosis (Figure 5 and Table 1). Additionally, ZnO-NPs markedly led to a slight decrease in the percentage of viable cells at 12, 24, and 48 hours of treatment. The result was similar to that of recently studied effects of nanoparticle on a human leukaemia cell lines* in vitro* done by Rahman et al. [33]. Thus, results suggested that the antiproliferative effect of ZnO-NPs toward WEHI-3 cells was via apoptosis.

### 3.6. Analysis of Cell Cycle Progression and Apoptosis by Flow Cytometry

**Figure 6:**
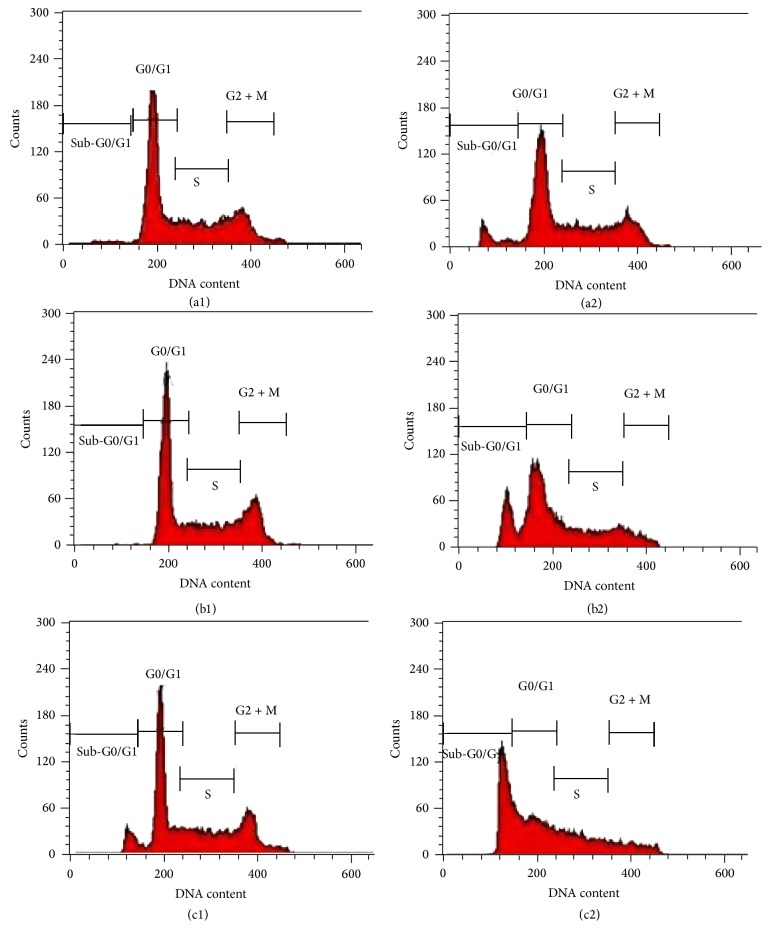
Cell cycle analysis of WEHI-3B cells treated with ZnO-NPs after staining with PI. (a1)–(c1) untreated WEHI-3B cells for 24, 48, and 72 h, respectively. (a2)–(c2) the effects of 24, 48, and 72 h, respectively, relative to exposure of WEHI-3B cells to ZnO-NPs. G0/G1, G2/M, and S indicate the cell phase, and Sub-G0-G1 refers to the apoptotic cells.

The stages of the cell cycle and the timing of the events at which the cell proliferates and divides were controlled and facilitated by various signalling pathways [35]. Effects of ZnO-NPs on cell cycle distribution were observed to determine the mechanism of its antiproliferative activity toward cancer cells. The exposure of WEHI-3 cells to growth suppressive of ZnO-NPs resulted in significant accumulation and distinct peak at sub-G0/G1, which represented the population of apoptotic and dead cells. Thus, the significant (*P* < 0.05) increase in sub-G1 population was time-dependent and ranged from 10.50 ± 0.28 after 24 hours to 22.00 ± 0.20 (48 hours) and to 25.85 ± 0.56 after exposure to ZnO-NPs for 72 hours. On the other hand, a clear decrease of cells with G0/G1 and G2/M DNA content was observed (Figure 6 and Table 2). These results suggested that ZnO-NPs could block the cell cycle and induce apoptosis and death in WEHI-3 cells time-dependently* in vitro*. Simultaneously, our results corroborated with those of Rahman et al. and Namvar et al. [33, 34].

### 3.7. Effect on Activation of Procaspase

**Figure 7:**
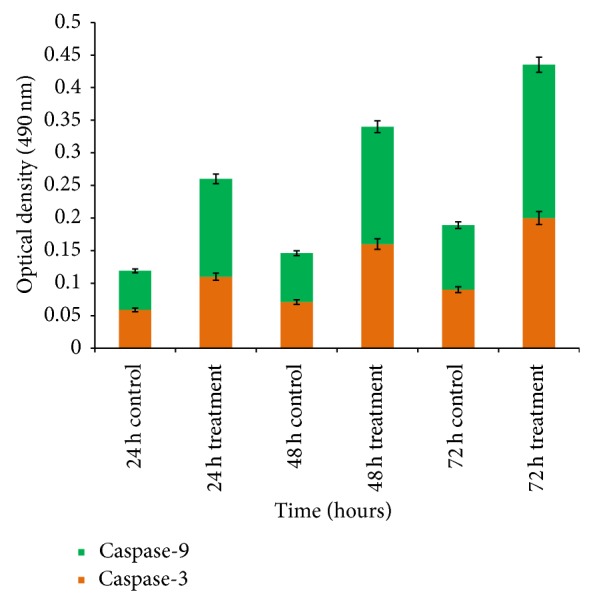
Effect of ZnO-NPs treatment on WEHI-3B cell caspase-3 and caspase-9. The values are mean % ± SD of three independent experiments. Significant differences (*P* < 0.05) between treated and control groups for caspase-3 and caspase-9 were found.

The anticancer effect of ZnO-NPs was further examined by quantifying the activities of caspase-3 and caspase-9 enzymes. Enzyme activities were evaluated in WEHI-3 cells treated with IC_50_ of ZnO-NPs for 24, 48, and 72 hours, respectively. The activity of caspase-3 and caspase-9 of the experimental groups and the control group was calculated. Results showed a significant (*P* < 0.05) increase of the enzyme activities time-dependently after exposure to ZnO-NPs for all treatment groups (Figure 7 and Table 3).

Caspases are a family of cysteine proteases that are known to form central parts of the apoptotic pathway. Caspase-3 and caspase-9 have been identified as key mediators of apoptosis in mammalian cells. Their activities are considered appropriate measures of cytotoxic responsiveness [36]. Thus, ZnO-NPs markedly activated an executioner caspase-3 and caspase-9 in a time-response fashion, which is consistent with the outcomes of other studies that have shown a number of prepared nanoparticles induce apoptosis through the activation of procaspases and the mitochondrial intrinsic pathway [33].

### 3.8. Effect on Apoptotic Proteins

**Figure 8:**
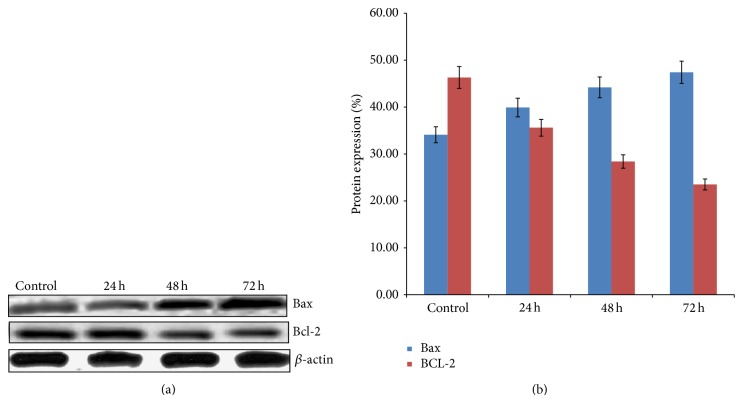
(a) Protein expression in treated WEHI-3B with ZnO-NPs for 24, 48, and 72 h observed by Western blotting assay. (b) Western blot transcription analysis of treated WEHI-3B with ZnO-NPs for 24, 48, and 72 h. Data analyzed using post hoc comparison of one-way ANOVA using Tukey's *b* test. The results showed significant (*P* < 0.05) Bax protein expressions and Bcl-2 protein suppression in all treated groups.

To detect the extent of ZnO-NPs on apoptosis-regulating proteins, the expression levels of both Bcl-2 and Bax proteins were investigated after incubation with ZnO-NPs in cultured WEHI-3 cells. Treatment with the ZnO-NPs significantly (*P* < 0.05) reduced the percentage (downregulated) of Bcl-2 protein expression in a time-dependent manner. In contrast, it significantly (*P* < 0.05) increased (upregulated) Bax expression (Figure 8).

Virtually, both proliferation and apoptosis are controlled by the mitochondrial pathway, which is mediated by the Bcl-2 family protein. High-expression of Bcl-2 protein has been found to protect cancer cells from apoptosis, whereas low Bax protein expression promotes cancer cells to undergo apoptosis. This effect is of high importance due to the fact that downregulation of Bcl-2 is associated with better therapeutic outcomes [37]. The mechanism of action of ZnO-NPs is unknown. However, different mechanisms of the anticarcinogenic activity of seaweed have been demonstrated. In addition, antiapoptotic activity of Bcl-2 has been found to be provoked by a homologous Bax protein that is able to form heterodimers with Bcl-2 [38, 39]. Thus, the ratio of Bcl-2 to Bax within the cell is the critical influential factor for the propensity of a cell to undergo apoptosis. The current study demonstrated that WEHI-3-ZnO-NPs treatment led to a decrease in Bcl-2 expression and an increase in the level of Bax, suggesting disruption of mitochondrial membranes. The decreased Bcl-2 levels along with increased levels of Bax may be sufficient to shift the balance toward apoptosis in WEHI-3 cells.

## 4. Conclusion

Based on the observations in this study, it was concluded that there was a time-dependent reduction of cell viability in treated cancer cells after exposure of ZnO-NPs, with no adverse effect on normal fibroblast cells. This provides new opportunities for the safe delivery and applications in anticancer therapy by ZnO-NPs. Moreover, ZnO-NPs demonstrated suppression in WEHI-3 cell growth and proliferation* in vitro*, which suggests that this is a potent and selective formula, and could be an alternative chemotherapeutic candidate for antileukemia in the near future. Collectively, our data suggested that ZnO-NPs provoked and induced apoptosis through an intrinsic mitochondrial pathway depending upon caspase activation.

## Figures and Tables

**Table 1:** Flow cytometric analysis of WEHI-3B cells after treated with ZnO-NPs. The cells were stained with FITC-conjugated annexin V and PI and incubated at 37°C for 12, 24, and 48 h, respectively.

Cells (%)						
Cell condition	Control 12 h	Treated 12 h	Control 24 h	Treated 24 h	Control 48 h	Treated 48 h
Viable cells	96.4 ± 0.75	80.59 ± 0.65	92.33 ± 0.55	78.29 ± 0.15	90.78 ± 0.25	73.5 ± 0.13
Early apoptosis	1.87 ± 0.15	8.75 ± 0.99^*^	2.98 ± 0.70	10.00 ± 0.30^*^	6.15 ± 0.45	15.5 ± 0.22^*^
Late apoptosis necrosis	1.69 ± 0.35	10.66 ± 0.95^**^	4.69 ± 0.50	11.7 ± 0.80^**^	3.07 ± 0.25	10.9 ± 0.10^**^

Values are expressed as mean ± SD of three different experiments. The data has been analyzed using post hoc comparison test one-way ANOVA; means were compared by Tukey's *b* test. ^*^Significant (*P* < 0.05) increased early apoptotic cells in ZnO-NPs treated groups compared to untreated controls. ^**^Significant (*P* < 0.05) increased late apoptotic/necrotic cells in ZnO-NPs-treated groups compared to untreated controls.

**Table 2:** Flow cytometric analysis of WEHI-3B cells after treated with ZnO-NPs. The cells were stained with PI and incubated at 37°C for 24, 48, and 72 h.

Cells (%)						
Cell cycle phases	Control 24 h	Treated 24 h	Control 48 h	Treated 48 h	Control 72 h	Treated 72 h
G0/G1	55.25 ± 0.06	33.40 ± 0.45	51.10 ± 0.29	42.61 ± 0.52	50.64 ± 0.32	37.68 ± 0.68
G2/M	18.85 ± 0.76	18.00 ± 0.41	19.16 ± 0.26	12.29 ± 0.35	9.30 ± 0.22	6.06 ± 0.93
Synthesis	24.91 ± 0.06	38.30 ± 0.33	29.24 ± 0.06	22.93 ± 0.12	37.50 ± 0.61	30.35 ± 0.18
Sub-G0/G1	0.9 ± 0.23	10.50 ± 0.28^*^	0.50 ± 0.34	22.00 ± 0.20^*^	2.20 ± 0.46	25.85 ± 0.56^*^

Values are expressed as mean ± SD of three different experiments. The data has been analyzed using post hoc comparison test one-way ANOVA; means compared with Tukey's *b* test. ^*^Significant (*P* < 0.05) increased cells in sub-G0/G1 phase in ZnO-NPs treated groups compared to untreated controls.

**Table 3:** Caspases spectrophotometric analysis of WEHI-3B cells after being treated with ZnO-NPs for 24, 48, and 72 h, respectively.

Cells %						
Caspase	Control 24 h	Treated 24 h	Control 48 h	Treated 48 h	Control 72 h	Treated 72 h
Caspase-3	0.059 ± 0.030	0.11 ± 0.01^*^	0.071 ± 0.051	0.16 ± 0.007^*^	0.09 ± 0.0032	0.2 ± 0.006^*^
Caspase-9	0.06 ± 0.071	0.15 ± 0.003^*^	0.075 ± 0.001	0.18 ± 0.005^*^	0.099 ± 0.002	0.235 ± 0.0035^*^

Values are expressed as mean ± SD of three different experiments. The data has been analyzed using post hoc comparison test one-way ANOVA; means compared with Tukey's *b* test. ^*^Significant (*P* < 0.05) increase of apoptotic cells in ZnO-NPs-treated groups compared with untreated controls.
